# Preoperative prognostic nutritional index is a powerful predictor of prognosis in patients with stage III ovarian cancer

**DOI:** 10.1038/s41598-017-10328-8

**Published:** 2017-08-25

**Authors:** Weiwei Zhang, Bin Ye, Weijiang Liang, Yazhou Ren

**Affiliations:** 1 Department of Medical Oncology, Nanfang Hospital, Southern Medical University, Guangzhou BaiYun Road 1838, 510515 Guangzhou, China; 2 Department of Medical Oncology, The Sixth People’s Hospital of Chengdu, 610051 Chengdu, China; 3 Big Data Research Center, School of Computer Science and Engineering, University of Electronic Science and Technology of China, 611731 Chengdu, China

## Abstract

Many established inflammation- and nutrition-related factors have been investigated as potential independent prognostic factors in various cancers, including the C-reactive protein/albumin ratio (CAR), lymphocyte/monocyte ratio (LMR), modified Glasgow prognostic score (mGPS), body mass index (BMI), and prognostic nutritional index (PNI). This study was performed to estimate the prognostic value of these factors in predicting survival and platinum resistance in ovarian cancer (OC), especially according to stage. Kaplan-Meier and multivariate analyses were performed to plot the survival curve and determine the independent prognostic factors. Additionally, the area under the receiver operating characteristic curve (AUC) was used to predict platinum resistance and prognosis by comparing the predictive ability of PNI and cancer antigen (CA)-125. In all patients, decreased PNI was significantly associated with platinum resistance and poor overall survival (OS) and progression-free survival (PFS). Regarding tumor stage, decreased PNI was significantly associated with poor PFS and OS only in stage III OC. Furthermore, the PNI also showed a significantly higher AUC value than CA-125 for predicting mortality and platinum resistance in all OC patients, but not in stage III patients. In conclusion, decreased PNI is a powerful predictor of a poor prognosis in OC, and especially for stage III cases.

## Introduction

In the United States, ovarian cancer (OC) is the leading cause of death from gynecologic cancer and is the fifth most common cause of cancer mortality in women. It has been estimated that there will be 22,440 new cases of, and 14,080 deaths from, OC in the United States in 2017, and less than 40% of affected women will be cured^1^. Recent data suggest that hormone therapy and pelvic inflammatory disease may increase the risk of OC^2,3^. Although most patients undergo primary cytoreductive surgery followed by chemotherapy, they always have a poor prognosis because of high chemotherapy resistance and advanced disease. Approximately 80% of OC patients will show tumor progression and relapse after first-line chemotherapy due to drug resistance or therapeutic failure within 1–2 years^4,5^. However, to our knowledge, the mechanism of chemoresistance remains unclear. It is still a major clinical challenge to clarify the relationships among inflammation, metabolism, drug resistance, and cancer, and to find an optimal prognostic factor for predicting chemotherapy resistance and survival of OC patients.

It has been found that preoperative nutritional and immunological condition, as well as systemic inflammatory response markers, are associated with the postoperative prognosis and overall survival (OS) of patients with malignant tumors^6^. Immune function can be affected by nutritional condition and inflammation status, which in turn affect the C-reactive protein (CRP) level and concentration of lymphocytes. Serum albumin, the synthesis of which is suppressed by malnutrition and inflammation, is generally used to assess nutritional status, severity of disease, disease progression, and prognosis^7^. In addition, albumin concentrations can be influenced by CRP concentrations, and this relationship is similar across various tumor types^8^. Therefore, in recent years, some inflammation- and nutrition-based factors have been investigated as possible prognostic and predictive markers in various cancers. Details of these factors are all easily available from peripheral blood samples, including the C-reactive protein/albumin ratio (CAR), lymphocyte/monocyte ratio (LMR), albumin and lymphocyte count combined into the prognostic nutritional index (PNI), and CRP- and albumin-related factors of the modified Glasgow prognostic score (mGPS)^9–12^. As an efficient, simple, and convenient novel prognostic factor, the PNI is calculated according to the following formula: serum albumin value (g/L) + 0.005 × lymphocyte count (per mm^3^) in peripheral blood^11^. Recently, PNI has been reported to be an independent prognostic factor for survival in different malignant carcinomas, including colorectal cancer, gastric cancer, lung cancer, and pancreatic cancer^13–16^. However, the prognostic importance of PNI for OC still needs to be elucidated, especially according to tumor stage. Although Miao *et al*.^17^ reported that PNI was an independent prognostic factor in OC patients, they did not assess the combination of PNI with other established prognostic factors, such as CAR, LMR, and mGPS. Thus, it is meaningful to combine the PNI and other established nutrition- and inflammation-related prognostic factors to obtain optimal independent prognostic scores for predicting the chemoresistance and clinical outcomes of OC patients at different stages.

## Results

### Patient characteristics

In total, 237 patients who were diagnosed with OC and underwent cytoreductive surgery followed by platinum-based chemotherapy were evaluated. Their median age was 50 years (range: 24–76 years). Among these patients, 131 (55.3%) were defined as platinum-sensitive and 106 (44.7%) were platinum-resistant. The majority of patients presented with serous ovarian carcinoma (n = 123, 51.9%) and were classified Federation of Gynecologists and Obstetricians (FIGO) stage III (n = 140, 59.1%) at initial diagnosis. The baseline characteristics of all patients are listed in Table 1.

**Table 1:** Correlations of PNI and clinicopathological characteristics of ovarian cancer patients.

Variable	n (%)	PNI		*P* value
		<47.2, n (%)	≥47.2, n (%)	
Age (years)				0.066
≤50	125 (52.7)	65 (47.4)	60 (60.0)	
>50	112 (47.3)	72 (52.6)	40 (40.0)	
Stage				<0.001
FIGO I	28 (11.8)	8 (5.8)	20 (20.0)	
FIGO II	39 (16.5)	15 (10.9)	24 (24.0)	
FIGO III	140 (59.1)	92 (67.2)	48 (48.0)	
FIGO IV	30 (12.7)	22 (16.1)	8 (8.0)	
Residual tumor mass				<0.001
≤2 cm	132 (55.7)	62 (45.3)	70 (70.0)	
>2 cm	105 (44.3)	75 (54.7)	30 (30.0)	
Histological subtype				0.001
Serous	123 (51.9)	61 (44.5)	62 (62.0)	
Non-serous	86 (36.3)	63 (45.9)	23 (23.0)	
Histological grade				0.237
G1	79 (33.3)	40 (30.0)	39 (39.0)	
G2	52 (21.9)	30 (21.9)	22 (22.0)	
G3	88 (37.1)	56 (40.9)	32 (32.0)	
Ascites				<0.001
Negative	102 (43.0)	34 (24.8)	68 (68.0)	
Positive	135 (57.0)	103 (75.2)	32 (32.0)	
CA-125 (U/ml)				<0.001
<35	26 (11.0)	5 (3.6)	21 (21.0)	
≥35	183 (77.2)	117 (85.4)	66 (66.6)	
Chemosensitivity				<0.001
Sensitive	131 (55.3)	61 (44.5)	70 (70.0)	
Resistant	106 (44.7)	76 (55.5)	30 (30.0)	
CAR				<0.001
<0.5	142 (59.9)	59 (43.1)	83 (83.0)	
≥0.5	95 (40.1)	78 (56.9)	17 (17.0)	
LMR				<0.001
<3.82	132 (55.7)	106 (77.4)	26 (26.0)	
≥3.82	105 (44.3)	31 (22.6)	74 (74.0)	
mGPS				<0.001
0	97 (40.9)	27 (19.7)	70 (70.0)	
1	76 (32.1)	46 (33.6)	30 (30.0)	
2	64 (27.0)	64 (46.7)	0 (0.0)	
BMI (kg/m^2^)				0.460
<18.5	31 (13.1)	20 (14.6)	11 (11.0)	
≥18.5	206 (86.9)	117 (85.4)	89 (89.0)	

CAR, C-reactive protein/albumin ratio; BMI, body mass index; FIGO, Federation of Gynecologists and Obstetricians; LMR, lymphocyte/monocyte ratio; mGPS, modified Glasgow prognostic score; PNI, prognostic nutritional index.

### Cutoff point for determining the PNI

**Figure 1:**
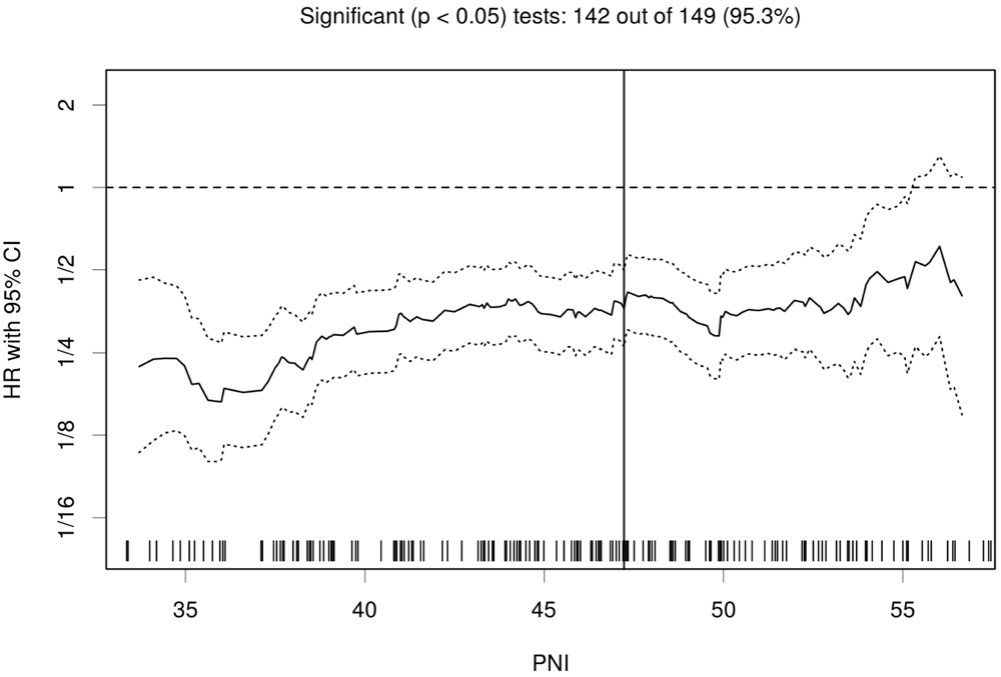
Hazard ratio (HR) for overall survival (OS) independent of the cutoff point for the prognostic nutritional index (PNI) in patients with ovarian cancer. The vertical line denotes the optimal cutoff point. The plots were generated using Cutoff Finder.

The median and mean levels of the PNI were 45.9 and 45.68 ± 7.14 (range: 26.60–65.60), respectively. Analyses using the biostatistical tool Cutoff Finder showed that a wide range of cutoff points for PNI were significant (Fig. 1). For OS, the optimal cutoff point of PNI was 47.2; patients were divided into two groups based on this cutoff value (PNI ≥ 47.2, n = 100, 42.2%; PNI < 47.2, n = 137, 57.8%).

### Correlation between PNI and clinicopathological parameters

The relation between preoperative PNI and the clinicopathological characteristics of patients with OC is shown in Table 1. Decreased PNI was significantly associated with advanced FIGO tumor stage (*P* < 0.001), maximum residual tumor (*P* < 0.001), histological subtype (*P* = 0.001), malignant ascites (*P* < 0.001), cancer antigen (CA)-125 ≥ 35 U/ml (*P* < 0.001), platinum resistance (*P* < 0.001), lower LMR (*P* < 0.001), and higher CAR (*P* < 0.001) and mGPS (*P* < 0.001). However, there were no significant associations between PNI and age (*P = *0.066), grade (*P = *0.237), or body mass index (BMI) (*P = *0.460). Among tumor stage III patients, decreased PNI was also significantly associated with residual tumor mass (*P = *0.023), histological subtype (*P = *0.005), malignant ascites (*P* < 0.001), CA-125 ≥ 35U/ml (*P* = 0.006), lower LMR (*P* < 0.001), and higher CAR (*P* < 0.001) and mGPS (*P* < 0.001), but not with platinum resistance (*P* = 0.095).

### Survival and prognostic factors

**Figure 2:**
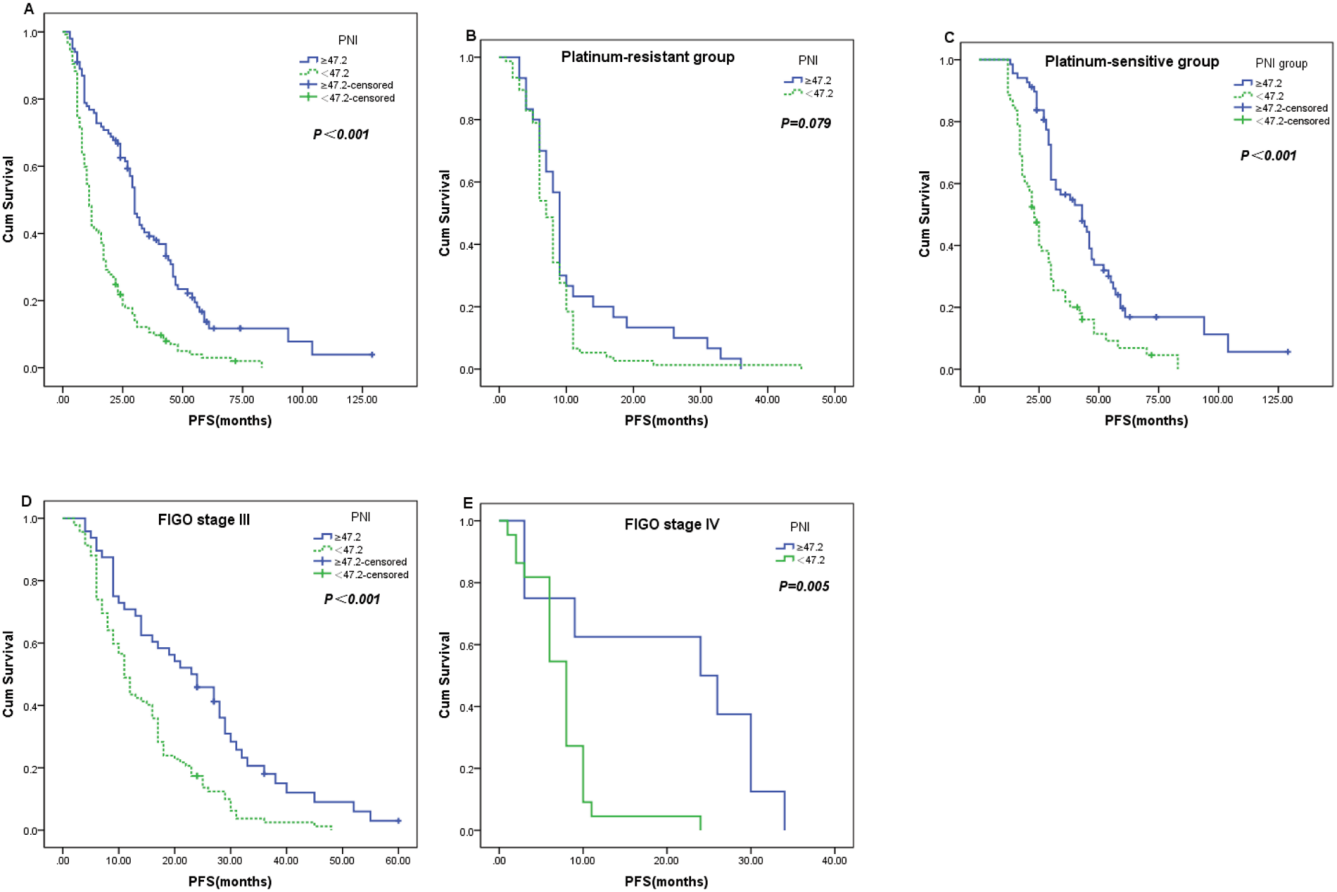
Kaplan–Meier progression-free survival curves showing the difference between the high- and low-PNI groups. (**A**) in all patients; (**B**,**C**) in platinum-resistant and platinum-sensitive subgroups; (**D**,**E**) in stage III and IV cases.

**Figure 3:**
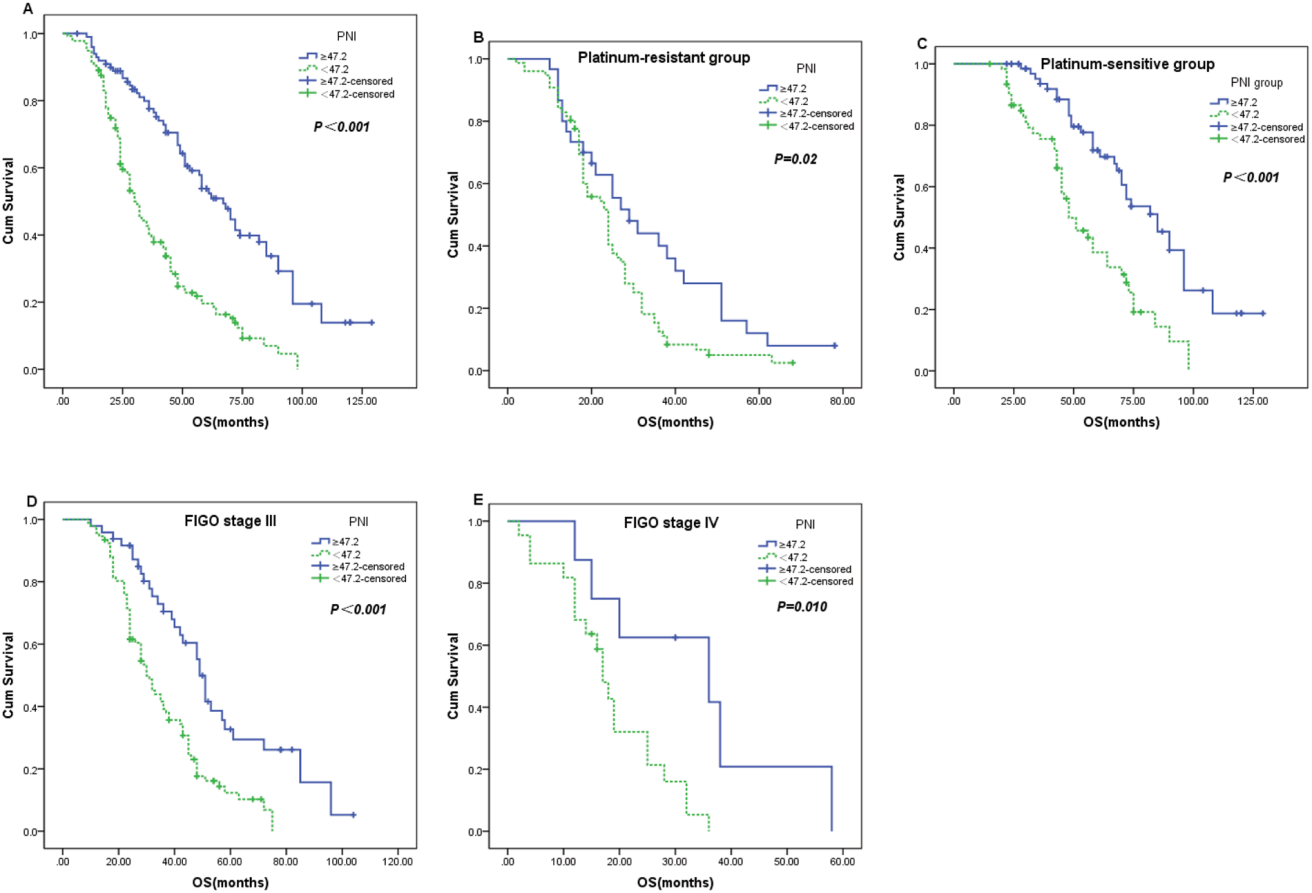
Kaplan–Meier OS curves showing the difference between the high-PNI and low-PNI groups. (**A**) in all patients; (**B**,**C**) in platinum-resistant and platinum-sensitive subgroups; (**D**,**E**) in stage III and IV cases.

The median progression-free survival (PFS) and OS in the whole study population were 17 months and 36 months, respectively. Patients in the low-PNI group had significantly shorter PFS (17.3 vs. 37.8 months, *P* < 0.001) and OS (38.7 vs. 68.8 months, *P* < 0.001) than those in the high-PNI group (Figs 2A and 3A). When the patients were divided into platinum-sensitive and -resistant groups, significant differences in PFS and OS between high- and low-PNI patients were observed only in the platinum-sensitive group (49.4 vs. 28.9 months, *P* < 0.001 and 55.7 vs. 82.7 months, *P* < 0.001 respectively), and not in the platinum-resistant group (11.3 vs. 8.0 months, *P* = 0.079 and 34.0 vs. 24.8 months, *P* = 0.020, respectively) (Figs 2B,C and 3B,C).

Univariate analyses revealed that FIGO tumor stage, residual tumor mass, massive ascites, CA-125 level, chemosensitivity, mGPS, PNI, CAR, LMR, and BMI were significantly associated with both PFS and OS (Tables 2 and 3). To assess the independent prognostic factors of OC, multivariate Cox proportional hazards were also assessed. In the multivariate Cox regression model, the FIGO tumor stage (hazard ratio [HR] = 1.478, 95% confidence interval [CI]: 1.170–1.868, *P* = 0.001), residual tumor mass (HR = 1.471, 95% CI: 1.009–2.144, *P* = 0.045), platinum resistance (HR = 7.427, 95% CI: 4.891–11.278, *P* < 0.001), mGPS (HR = 1.327, 95% CI: 1.053–1.673, *P* = 0.017), PNI (HR = 2.096, 95% CI: 1.380–3.185, *P* = 0.001), and BMI (HR = 1.828, 95% CI: 1.155–2.895, *P* = 0.010) were significantly associated with PFS (Table 2). However, only FIGO tumor stage (HR = 1.933, 95% CI: 1.461–2.556, *P* < 0.001), platinum resistance (HR = 4.832, 95% CI: 3.213–7.266, *P* < 0.001), and PNI (HR = 2.544, 95% CI: 1.761–3.675, *P* < 0.001) were also independently and significantly associated with shortened OS (Table 3).

**Table 2:** Univariate and multivariate Cox proportional hazards analysis of progression-free survival.

Variable	HR	Univariate	*P* value	HR	Multivariate	*P* value
		95% CI			95% CI	
Age (years) (≤50 vs. >50)	0.990	0.756–1.298	0.945			
FIGO stage (I/II/III/IV)	2.390	1.966–2.907	<0.001	1.478	1.170–1.868	0.001
Histological grade (G1/G2/G3)	1.146	0.973–1.350	0.102			
Histological subtype (serous vs. non-serous)	1.075	0.802–1.441	0.629			
Residual tumor mass (≤2 cm vs. >2 cm)	3.500	2.582–4.744	<0.001	1.471	1.009–2.144	0.045
Ascites (yes vs. no)	2.127	1.603–2.821	<0.001			
CA-125 (U/ml) (<35 vs. ≥35)	2.025	1.267–3.236	0.003			
Chemosensitivity (sensitive vs. resistant)	8.390	6.066–11.605	<0.001	7.427	4.891–11.278	<0.001
mGPS (0/1/2)	1.750	1.470–2.083	<0.001	1.327	1.053–1.673	0.017
PNI (<47.2 vs. ≥ 47.2)	2.411	1.807–3.216	<0.001	2.096	1.380–3.185	0.001
CAR (<0.5 vs. ≥ 0.5)	1.751	1.326–2.312	<0.001			
LMR (<3.82 vs. ≥ 3.82)	2.271	1.702–3.030	<0.001			
BMI (<18.5 vs. ≥ 18.5)	2.615	1.746–3.917	<0.001	1.828	1.155–2.895	0.010

CAR, C-reactive protein/albumin ratio; BMI, body mass index; FIGO, Federation of Gynecologists and Obstetricians; LMR, lymphocyte/monocyte ratio; mGPS, modified Glasgow prognostic score; PNI, prognostic nutritional index.

**Table 3:** Univariate and multivariate Cox proportional hazards analysis of overall survival.

Variables	HR	Univariate	*P* value	HR	Multivariate	*P* value
		95% CI			95% CI	
Age (years) ≤50 vs. >50)	1.002	0.742–1.353	0.990			
FIGO stage (I/II/III/IV)	2.781	2.183–3.543	<0.001	1.933	1.461–2.556	<0.001
Histological grade (G1/G2/G3)	1.151	0.955–1.388	0.139			
Histological subtype (serous vs. non-serous)	1.276	0.923–1.762	0.140			
Residual tumor mass (≤2 cm vs. >2 cm)	3.349	2.405–4.664	<0.001			
Ascites (negative vs. positive)	2.331	1.693–3.211	<0.001			
CA-125 (U/ml) (<35 vs. ≥35)	2.299	1.299–4.067	0.004			
Chemosensitivity (sensitive vs. resistant)	5.965	4.235–8.402	<0.001	4.832	3.213–7.266	<0.001
mGPS (0/1/2)	1.652	1.360–2.008	<0.001			
PNI (<47.2 vs. ≥47.2)	2.701	1.946–3.749	<0.001	2.544	1.761–3.675	<0.001
CAR (<0.5 vs. ≥0.5)	1.763	1.300–2.390	<0.001			
LMR (<3.82 vs. ≥3.82)	2.670	1.938–3.678	<0.001			
BMI (<18.5 vs. ≥18.5)	1.842	1.181–2.874	0.007			

CAR, C-reactive protein/albumin ratio; BMI, body mass index; FIGO, Federation of Gynecologists and Obstetricians; LMR, lymphocyte/monocyte ratio; mGPS, modified Glasgow prognostic score; PNI, prognostic nutritional index.

When patients were stratified by FIGO tumor stage, high-PNI patients had significantly longer PFS than low-PNI patients only for cases of FIGO tumor stages III (*P* < 0.001) and IV (*P* = 0.005) (Fig. 2D,E). Similarly, high-PNI patients had significantly longer OS than low-PNI patients only in stages III (*P* < 0.001) and IV (*P* = 0.010) (Fig. 3D,E). However, the multivariate Cox regression model demonstrated that the PNI was an independent predictive factor of poor PFS (HR 1.815, 95% CI 1.113–2.958, *P* = 0.017) and OS (HR 1.699, 95% CI 1.035–2.789, *P* = 0.036) only in FIGO tumor stage III OC patients, as were residual tumor mass and chemosensitivity. All these findings show that the PNI is an independent risk factor for poor PFS and OS in OC patients, especially those at stage III.

### Comparison of predictive ability

**Figure 4:**
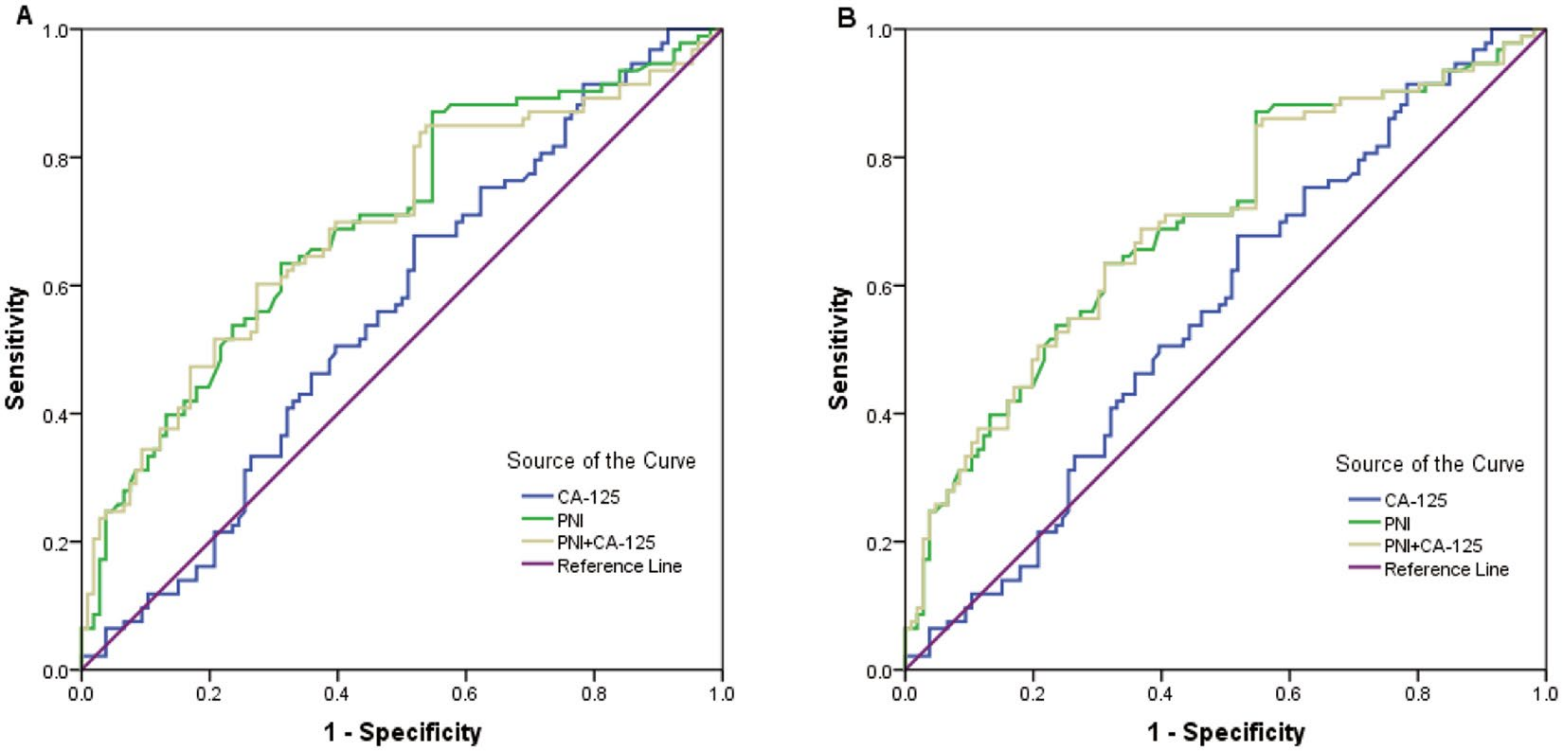
Receiver operating characteristic curves of PNI, CA-125, and both in combination with respect to the prediction of OS (**A**) and platinum resistance (**B**).

The receiver operating characteristic curve (ROC) and area under the receiver operating characteristic curve (AUC) values were used to compare the predictive ability among CA-125, PNI, and their combination for OS and platinum resistance (Fig. 4 and Table 4). With respect to predicting mortality, the PNI had a significantly higher AUC value than the CA-125 (0.677 vs. 0.567, *P* = 0.044). The combination of the PNI and CA-125 had a higher AUC value than either alone, although the difference was not significant (*P* > 0.05). Regarding platinum resistance, the PNI showed a higher AUC value than CA-125 (0.699 vs. 0.560, *P* = 0.006) and their combination (0.699 vs. 0.692, *P* = 0.847). The combination of the PNI and CA-125 had a significantly higher AUC value than CA-125 (*P* = 0.007). However, the PNI did not have a significantly higher AUC than CA-125 with respect to OS (0.649 vs. 0.547, *P* = 0.388) or platinum resistance (0.618 vs. 0.520, *P* = 0.094) among FIGO tumor stage III patients. Furthermore, the combination of PNI and CA-125 also did not have significantly higher AUC value than either one alone.

**Table 4:** Comparison of the diagnostic performance in predicting mortality and chemoresistance.

Variable	Overall survival		Platinum resistance	
	AUC(95% CI)	*P*	AUC(95% CI)	*P*
CA-125	0.567 (0.466–0.669)	0.152	0.560 (0.480–0.639)	0.146
PNI	0.677 (0.598–0.755)	<0.001	0.699 (0.632–0.765)	<0.001
CA-125 + PNI	0.688 (0.600–0.776)	<0.001	0.696 (0.623–0.770)	<0.001

AUC, area under the receiver operating characteristic curve; PNI, prognostic nutritional index.

## Discussion

To date, no widespread nutrition- or inflammation-related factor has been found to index chemoresistance or prognosis in OC patients, especially according to tumor stage. Although the association between PNI and prognosis has been clarified in other cancers^18^, the impact of PNI on platinum resistance and clinical outcomes in OC, especially according to tumor stage, has not been clarified.

Laky *et al*.^19^ showed that about 20% of newly diagnosed gynecologic cancer patients have malnutrition. More than 20% of cancer patients die from malnutrition rather than the cancer itself^20^. Due to the metabolic effects of tumor mass, malignant ascites, and small bowel obstruction, OC patients are more likely to present with malnutrition and cachexia^21^. Furthermore, the tumor is more prone to develop chemoresistance in malnourished OC patients^22^. Recently, Matassa *et al*.^23^ also observed that oxidative metabolism drives inflammation-induced platinum resistance in OC. Lymphocytes were also reported to play a major role in immune responses by mediating the immunologic damage caused by various cancers^24^. As components of the PNI, both the albumin count and the lymphocyte count are closely related to inflammatory responses in cancer patients, which are independent predictors of long-term outcomes in OC^25,26^. According to the prognostic association between PNI and albumin and lymphocyte counts, it seems that PNI is a reflection of systemic inflammation, which may influence cancer growth and metastasis^17^. Thus, both inflammation- and malnutrition-related prognostic factors may induce chemotherapy resistance and predict the OS of OC patients.

Consistent with previous studies, our study demonstrated that FIGO tumor stage was an independent prognostic factor in OC patient^27^. To estimate the clinical outcomes of OC patients better, many inflammation- and malnutrition-based markers, such as BMI, LMR, mGPS, and CAR, have been investigated as potentially important prognostic and predictive factors in OC patients^28–30^. Similar to the study by Miao *et al*.^17^ our study demonstrated that the independent prognostic factor best predicting the OS of OC patients was PNI rather than BMI, CAR, LMR, or mGPS. The chi-square test determined that a PNI < 47.2 was not only associated with advanced FIGO tumor stage, maximum residual tumor, malignant ascites, platinum resistance, and lower LMR but also with higher CAR and mGPS. However, our study further showed that when patients were stratified by FIGO tumor stage, stage III patients showed the most significant association between PNI level and the outcome of the disease. Furthermore, ROC and AUC analyses showed that PNI was significantly superior to CA-125 in predicting mortality and platinum resistance in all-stage OC patients, but not in stage III cases. These results suggest that as an easily available laboratory hematological marker, PNI is superior to other nutrition- and inflammation-related prognostic factors in predicting survival in OC patients, especially for FIGO tumor stage III patients. Furthermore, PNI may also predict the platinum-based chemotherapeutic response of all-stage OC patients.

This study provides further support for the proposition that elevated preoperative PNI is associated with a good prognosis in OC patients. A study by Liu *et al*.^31^ showed that the CAR had superior prognostic ability compared to other established inflammation-related prognostic indices, such as the PNI, mGPS, neutrophil/lymphocyte ratio (NLR), and platelet/lymphocyte ratio (PLR) in 200 OC patients. The reason for this difference may be that our study included LMR, BMI, and platinum resistance. In addition, the current study not only further assessed the correlation between PNI and tumor stage but also compared the predictive ability of CA-125, the PNI, and their combination with respect to OS and platinum resistance, according to ROC and AUC values. Nevertheless, both Liu *et al*.^31^ and the present study used retrospective, single-center studies, and the number of patients was small in both. Therefore, more studies are needed to confirm these results. Furthermore, the mechanisms linking the PNI, poor prognosis and platinum resistance must be clarified.

## Materials and Methods

### Patients

In total, 237 newly diagnosed OC patients, treated with cytoreductive surgery and platinum-based chemotherapy between January 2007 and December 2015 at Nanfang Hospital of Southern Medical University, were identified. Pathological parameters, clinical data, and survival times were extracted from medical records. Patients who had active infection, coexisting hematologic malignancies, or other hematologic or autoimmune disorders were excluded. The primary endpoint of the study was PFS, which was calculated from the date of treatment to the date of recurrence or progression. OS was defined as the time from treatment to the date of death or last follow-up. All OC patients were followed up every 2–4 months for the first 2 years, and every 3–6 months thereafter until December 2016. At each visit, the patients were assessed by clinical and imaging examinations and the serum levels of CA-125 of patients were assessed. This study was approved by the medical ethics committee of Southern Medical University. All methods were performed in accordance with the relevant guidelines and regulations. Written informed consent was obtained from each patient.

All of the following data were obtained from medical records: age, BMI, FIGO stage, massive ascites, surgery, residual tumor mass, tumor (histology, grade), chemosensitivity, and clinical characteristics (CA-125, CRP, albumin, lymphocyte, and monocyte levels). Based on previous studies, optimal debulking was defined as a maximum diameter of residual tumor after surgery of ≤2 cm^32,33^. Patients were defined as platinum-resistant if the disease progressed within 6 months after completing first-line platinum-based chemotherapy, while all other patients were defined as platinum-sensitive^34^. PNI was calculated according to the following formula: serum albumin (g/L) + 0.005 × lymphocyte count (per mm^3^) in the peripheral blood^11^. CAR was calculated by CRP (mg/L)/albumin (g/L) ratio^35^. LMR was defined as the absolute lymphocyte count/absolute monocyte ratio^36^. The mGPS encompassed both the CRP and albumin concentrations. Patients with both CRP > 10 mg/L and albumin < 35 g/L were allocated a score of 2. Patients with both CRP ≤ 10 mg/L and albumin ≥ 35 g/L were allocated a score of 0. Patients with only one of these abnormal levels were given a score of 1^12^. BMI, CAR, and LMR were categorized into two groups according to the cutoff values of ≥18.5 kg/m^2^, ≥0.5, and ≥3.82, respectively^9,37,38^.

### Statistical analysis

Statistical analyses were performed with SPSS software (ver. 20.0; IBM Corp., Armonk, NY, USA). Comparisons between categorical variables were performed using the chi-square test. The optimal cutoff value for PNI was determined via a web-based application, programmed in R by Budczies *et al*. (http://molpath.charite.de/cutoff/)^39^. Significant prognostic variables in univariate analyses were included in multivariate Cox regression models to determine independent prognostic factors, using a forward stepwise method. Differences in survival among classification groups were analyzed using Kaplan-Meier curves and log-rank tests. ROC curves were calculated for PNI and CA-125, alone and in combination. The AUC values were compared using MedCalc software (ver. 15.2.1; MedCalc Software bvba, Ostend, Belgium). A two-sided *P* value < 0.05 was considered statistically significant.
