# Author Correction: Preoperative prognostic nutritional index is a powerful predictor of prognosis in patients with stage III ovarian cancer

**DOI:** 10.1038/s41598-018-27841-z

**Published:** 2018-06-22

**Authors:** Weiwei Zhang, Bin Ye, Weijiang Liang, Yazhou Ren

**Affiliations:** 1 Department of Medical Oncology, Nanfang Hospital, Southern Medical University, Guangzhou BaiYun Road 1838, 510515 Guangzhou, China; 2 Department of Medical Oncology, The Sixth People’s Hospital of Chengdu, 610051 Chengdu, China; 3 Big Data Research Center, School of Computer Science and Engineering, University of Electronic Science and Technology of China, 611731 Chengdu, China

Correction to: *Scientific Reports* 10.1038/s41598-017-10328-8, published online 25 August 2017

In the original version of this Article, Affiliations 1, 2 and 3 were not listed in the correct order. The correct affiliations are listed below:

Affiliation 1:

Department of Medical Oncology, Nanfang Hospital, Southern Medical University, Guangzhou BaiYun Road 1838, 510515, Guangzhou, China

Affiliation 2:

Department of Medical Oncology, The Sixth People’s Hospital of Chengdu, 610051, Chengdu, China

Affiliation 3:

Big Data Research Center, School of Computer Science and Engineering, University of Electronic Science and Technology of China, 611731, Chengdu, China

Additionally, the original version of this Article omitted an affiliation for Weiwei Zhang. The correct affiliations for Weiwei Zhang are listed below:

Department of Medical Oncology, Nanfang Hospital, Southern Medical University, Guangzhou BaiYun Road 1838, 510515, Guangzhou, China

Department of Medical Oncology, The Sixth People’s Hospital of Chengdu, 610051, Chengdu, China

Finally, in the original version of this Article, Weiwei Zhang was incorrectly listed as the corresponding author. The correct corresponding authors for this Article are Weijiang Liang and Yazhou Ren. Correspondence and requests for materials should be addressed to W.L. (email: wjliang22@126.com) or Y.R. (email: Yazhou.ren@uestc.edu.cn).

These errors have now been corrected in the PDF and HTML versions of the Article.

